# Digital finance, innovation transformation, and resilient city growth

**DOI:** 10.1038/s41598-024-56998-z

**Published:** 2024-03-25

**Authors:** Wenwu Zhang, Jinguo Wang, Hongkui Jin

**Affiliations:** 1 College of Economics and Management, Nanjing Forestry University, Long Pan Road No.159, Nanjing, 210037 Jiangsu China; 2 Nanjing University of Finance and Economics, Nanjing, China

**Keywords:** Digital finance, Resilient cities, Innovation transformation, Digital governance, Engineering, Mathematics and computing

## Abstract

Digital finance is a pivotal catalyst for a contemporary economic system and assumes a significant auxiliary function in the establishment of resilient urban centers. This study empirically examines the enabling influence of digital finance on resilient cities using panel data from 287 prefecture-level cities and above in China between 2011 and 2020. The analysis is based on the mechanisms of innovation and transformation. The importance of digital finance in facilitating the development of resilient cities has been observed, with a specific emphasis on its impact on enhancing the adaptive capacity and growth resilience of urban areas. The utilization of digital finance has the potential to expedite the process of transforming urban industrial structures, invigorating innovation and entrepreneurial activities, and serving as a significant catalyst for the development of resilient cities. The analysis of heterogeneity reveals that various aspects of digital finance have varying degrees of influence on urban resilience. Specifically, the depth of utilization of digital finance exerts the most significant impact, followed by the level of digitalization, while the extent of coverage has the least effect. Furthermore, when considering regional distribution, the promotion effect of digital finance on resilient cities diminishes gradually from the eastern to the central and western regions.

## Introduction

In the present-day context, urban development is encountering a growing array of intricate and multifaceted hazards and obstacles. The occurrence of unpredictable shocks to urban areas, ranging from natural disasters and public emergencies to socio-economic swings, is increasingly frequent. Historically, urban areas have addressed these difficulties by enhancing emergency preparedness strategies and risk mitigation measures. However, this responsive governance approach is no longer satisfactory for effectively addressing multifaceted and evolving urban concerns. Enhancing urban governance and fostering sustainable development capacities, as well as establishing resilient cities, have emerged as vital strategies for mitigating the repercussions of unpredictable shocks and attaining development of superior quality. The report presented at the 20th National Congress of the Communist Party of China outlines the objective of enhancing urban planning, building, and governance, as well as the aspiration to establish livable, resilient, and smart cities. This entails imposing more stringent criteria for urban development and construction. Within this particular context, the examination of methods to improve urban resilience and construct cities that are robust holds considerable academic worth and significance.

Urban resilience, in an economic context, typically pertains to the capacity of cities to recover from external shocks and effectively mitigate, respond to, and recuperate from severe disasters. This ability is contingent upon the interplay of various factors that contribute to urban development. Prior research has indicated that financial development, as a type of high-end producer service, plays a significant role in facilitating urban modernization and industrial upgrading^1^^,^^2^. Digital finance has a crucial role in fostering the resilient growth of cities, as it emerges as a prominent economic form and a driving factor for urban advancement. The emergence of digital finance has led to the provision of financial services that are more efficient and easy. This has resulted in increased fluidity and interactivity of urban resources and markets, hence enhancing their ability to address the difficulties posed by urban multi-risks. For instance, the utilization of digital finance has the potential to enhance credit risk management in urban areas by employing intelligent risk control models. Additionally, it can offer digital financial remedies to effectively address natural calamities, diseases, and other unforeseen emergencies, thereby ensuring public safety and facilitating sustainable development in the future^3^^,^^4^. In a hypothetical scenario, if a particular region possesses a robust financial system and acknowledges the significant influence of market mechanisms in the allocation of financial resources, the utilization of digital finance during times of uncertainty in cities can facilitate prompt adaptive modifications, mitigate and address risks, and ultimately foster resilient development. Nevertheless, China's urban growth exhibits notable regional disparities, characterized by insufficient digital infrastructure, poor market regulation mechanisms, and large financial supply and demand tensions across different regions. In the present climate, is it feasible for digital finance to provide assistance in fostering resilient urban areas? What are the precise impacts? Regrettably, the current body of literature pertaining to the aforementioned topics remains constrained in terms of both coverage and depth of study. To establish resilient cities that rely on digital money, it is imperative to develop a comprehensive institutional framework and conduct more meticulous empirical investigations. Within this context, the present article directs its attention towards examining the connection and function of digital finance in the context of resilient cities. It undertakes an analysis of the mechanisms and impact of digital finance on the resilience of cities while also putting forth suggestions on how digital transformation and urban innovation capabilities can effectively enhance urban resilience. By doing so, this article aims to offer valuable references and insights for the comprehensive integration and mutual advancement of digital finance and urban resilience.

There are three main branches of literature related to this article. The first literature mainly focuses on the definition, measurement, and influencing factors of urban resilience. Scholars Reggiani et al.^5^ first introduced resilience into the study of spatial economics, defining it as the ability to respond in a timely manner and quickly make adjustments to return the economy to normal track. In subsequent research, more scholars have drawn on the theory of evolutionary resilience, paying more attention to the dynamic process of urban economic systems, arguing that urban resilience should include four aspects: resistance, resilience, adaptability, and renewal^6^, and a variety of measurement methods have emerged. There are two main methods for measuring urban resilience that are commonly used in practical research. The first method calculates the level of urban resilience by selecting a series of indicators and assigning them a certain weight^7^. For example, Martin et al.^8^ selected indicators from four dimensions, including urban resistance, resilience, readjustment capacity, and the ability to create economic growth paths, to measure the level of urban resilience, Chinese scholars Sun Jiuwen and Xu Yuan also mostly agree with Martin’s above-mentioned views and practices, and choose corresponding indicators based on China's actual situation to conduct relevant research, and have obtained research conclusions that are in line with the actual situation^9^. The latter method characterizes the changes in regional GDP, unemployment, and the difference between the national indicators during the same period^10,11^. In the research on factors affecting urban resilience, most literature mainly focuses on industrial structure, innovation and entrepreneurship, labor skills, and economic agglomeration^12–15^. The second literature focuses on the impact of digital finance on economic growth. Previous studies have shown that the development of digital finance has a significant positive effect on alleviating financing constraints, enhancing regional innovation and entrepreneurship activity, promoting household consumption, and reducing poverty, ultimately driving economic growth^16–19^. In addition, some scholars have also focused on the impact of digital finance on regional economic performance, pointing out that digital finance can improve innovation levels and the degree of regional opening up by alleviating credit constraints for enterprises, and fully leverage the inclusive attributes to benefit more groups from development outcomes, achieving high-quality economic development^20,21^. The third branch of literature most closely related to this study mainly focuses on the impact of digital finance on urban resilience. The inclusiveness brought by financial development can be enjoyed through the use of savings, credit, insurance, and digital payment products, which is reflected in the improvement of the availability of financial services. When the economy suffers from external shocks, it is beneficial to reduce economic fluctuations and stabilize the financial system^22^. As a cutting-edge financial form, digital finance has a very obvious inclusive development characteristic, which can not only directly affect the resilience level, but also indirectly affect the resilience level by enhancing entrepreneurial activity, narrowing the urban–rural income gap and promoting the upgrading of industrial structure, and can also drive the improvement of economic resilience level in surrounding areas through positive spatial spillover effects^23–25^. In addition, digital finance has continuously innovated and launched financial products through the establishment of online financial platforms, which has improved the level of social security while also increasing household property income. Social security capabilities are significantly positively correlated with cities' ability to resist external shocks and adjust and recover^26,27^. Recently, some Chinese scholars have begun to pay attention to the importance of green development, and have innovated to integrate digital finance, green innovation, and urban resilience into a framework for analysis, and the research shows that digital finance and green innovation can have synergistic and mutually reinforcing effects, thereby improving urban resilience and helping cities achieve sustainable development^28^. On this basis, the importance of green governance is further analyzed, and the development of an environment-friendly economy and the promotion of green governance are urgently emphasized^29^.

The above-mentioned literature has explored the relationship between digital finance and urban resilience to a certain extent, and has accumulated rich supporting literature. However, there are still some areas that deserve further expansion. First, existing research mainly discusses the relationship between digital finance and urban resilience from a holistic and comprehensive perspective, and the theoretical analysis of the mechanism between the two is not detailed enough. Secondly, the current literature on digital finance or resilience adopts a wide range of samples, mostly from provincial-level regions, and the evidence at the city level and above is still insufficient. Therefore, this article focuses on theoretical discussions and empirical analysis of digital finance, innovation transformation, and resilient cities, providing more substantial research evidence.

Compared with existing literature, the main innovations and contributions of this article are reflected in the following three aspects: First, from the perspective of research, this article analyzes the mechanism of digital finance in promoting industrial transformation and innovation and entrepreneurship, and constructs an economic analysis framework for digital finance to enhance urban resilience, enriching the relevant literature on the study of high-quality urban economic development. Secondly, in terms of research samples, this article uses economic data from cities at the prefecture level and above in China to construct resilience city measurement indicators, and matches them with digital finance indexes, extending the data dimension of existing empirical samples and providing more detailed research evidence. Thirdly, in terms of policy implications, Considering the success of China’s digital finance development, it provides valuable experience for the world, especially developing countries, and provides a new policy basis for further guiding the sound and healthy development of global digital finance, improving digital governance capabilities and economic resilience, and helping global economic recovery.

The remaining content structure of this article is arranged as follows: the second part is the background and theoretical analysis; the third part is the research design; the fourth part is the empirical result analysis; the fifth part is the heterogeneity test and mechanism analysis; and the sixth part is the basic research conclusion and relevant policy recommendations.

## Theoretical mechanisms and research hypotheses

The global financial industry has increasingly focused on digital finance as a key area of development, owing to the ongoing progress in digital transformation and innovation. Digital finance leverages sophisticated information technology to deliver financial services that are more efficient, convenient, and safe for both individuals and enterprises. This is achieved through the introduction of novel financial products and services. The ramifications of digital finance on economic growth and social development have been significant. The development of digital finance not only brings new business opportunities and a competitive landscape but also poses new challenges to the innovative transformation and resilient development of cities^30^. Cities have a significant role in facilitating the adoption and implementation of digital finance, as they serve as the primary drivers of economic and social progress. Digital finance offers enhanced efficiency, convenience, and security in the provision of financial services to urban areas, fostering market openness and resource liquidity. Simultaneously, the digitalization of urban areas and their capacity for innovation also serve as crucial facilitators and assurances for the expeditious advancement of digital finance. The primary distinction between digital finance and traditional finance is the widespread utilization of digital technologies, including the Internet, cloud computing, artificial intelligence, and blockchain, within the realm of digital finance. Digital finance has emerged as a transformative force in the financial industry, facilitated by online financial platforms. By using the power of technology, digital finance overcomes the constraints of time and place, enabling all microeconomic entities to access financial services on an equal footing. Consequently, this innovative approach efficiently addresses the long-tail conundrum. Simultaneously, by leveraging digital technology, digital finance effectively facilitates the movement of financial resources across regions, diminishes production and operational expenses, streamlines information retrieval, efficiently consolidates productive service resources, fosters the integration of industrial chains, and achieves the judicious allocation of resources in all facets of production. Consequently, this expedites the process of digital transformation.

Additionally, digital finance has the potential to guide residents' consumption and stimulate domestic demand. Consumption, being one of the three primary catalysts for economic expansion, possesses inherent benefits in contrast to investment, which relies on borrowing and entails a time lag before yielding outcomes, and export, which is heavily reliant on foreign demand. The role of consumption is poised to emerge as the most important engine for fostering economic expansion. The research revealed that digital finance exerts a double influence on the rise of consumption and the process of upgrading. Digital finance has the potential to enhance the convenience of payment and remove spatial and temporal limitations on residents' consumption. This can result in improved efficiency of residents' consumption and a boost in their consumption expenditure, ultimately promoting the growth of residents' consumption. Consumption growth has not only directly contributed to economic expansion but has also virtually augmented government fiscal revenue. The allocation of fiscal resources by the government has been directed towards various sectors, including improving people's livelihoods, enhancing employment opportunities, and fostering innovation. These efforts aim to reduce the income disparity between urban and rural populations, elevate the employment rate, stimulate innovation and entrepreneurship, drive industrial technological advancements, facilitate high-quality growth in regional economies, and bolster overall economic resilience. In contrast, digital finance has the potential to mitigate the limitations associated with capital credit, thereby leading to an augmentation in consumers' expenditures beyond their basic survival needs. The transformation of survival material consumption into a source of enjoyment has the potential to stimulate individuals' inventive consciousness, fulfill a wider range of consumer needs, facilitate consumption upgrading, and ultimately contribute to economic growth.

Furthermore, digital finance can promote the digital innovation and transformation of enterprises. As an important part of the market economy, small and medium-sized enterprises play an important role in stabilizing growth, promoting reform, adjusting structure, benefiting people's livelihood and preventing risks. However, small and medium-sized enterprises often suffer discrimination in the financing process, which makes small and medium-sized enterprises that really need financial support to innovate and transform into the dilemma of not daring to innovate and not being able to innovate. Digital finance can use advanced digital technology to accurately estimate and profile enterprises in the economic system, objectively and accurately evaluate enterprise users, realize the maximum matching of financial resources and financial needs of enterprises, and provide stable capital flow and high-quality and efficient financial services for small and medium-sized enterprises. At the same time, advanced financial supervision methods relying on digital technology can also monitor and feedback risk information in a timely manner, and use credit, insurance and other financial means to hedge risks, improving the economic system's risk tolerance and response speed, thereby making the economic system more stable. Based on the aforementioned analysis, this article posits its initial hypothesis:

### H1

The implementation of digital finance has the potential to significantly enhance the level of resilience in urban areas, hence facilitating the development of resilient cities.

Based on the Kuznets Law, it may be posited that with economic expansion, there is a corresponding increase in the absorption of labor by the service industry, underscoring its notable employment flexibility and growing significance. According to Lin^31^, industrial structure transformation plays a crucial role in urban resource allocation, economic growth, and the enhancement of resource value. It is seen as the central focus for optimizing the economic structure of urban areas. Different types of industries exhibit varying demand elasticities, distinct export orientations, diverse levels of labor and capital intensity, as well as differing levels of external competitive risk. Regions with overly homogeneous industrial structures, or those overly reliant on a cluster of interlinked local industries within the supply chain, are more susceptible to external shocks^32^. A rationalized, modern, and diversified industrial structure possesses the capacity to effectively demonstrate its automatic stabilizer attributes in the face of external shocks, therefore mitigating economic fluctuations, diversifying risks, and diminishing uncertainty. The process of industrial structural transformation has the potential to facilitate the shift from an extensive to an intensive economic growth mode by means of technological innovation and the transfer of labor production factors. This transformation can lead to improved efficiency in resource utilization, ultimately resulting in high-quality economic development and strengthened urban resilience.

Digital finance can leverage its inherent advantages to influence the transformation of industrial structures in the following ways: (1) The advancement of digital finance has reduced the degree of information asymmetry between borrowers and lenders, as well as within industries. It has broadened enterprises' financing channels and lowered the cost of capital^33,34^. This has improved the financing environment for traditional industries and marginalized groups, propelling the digital transformation of conventional sectors and achieving a highly polarized and rationalized industrial structure. (2) Digital finance, bolstered by robust digital technology, expedites the leapfrog development of high-tech industries, surmounting early-stage obstacles. It facilitates the diffusion and implementation of cutting-edge technologies across industries and society, yielding technological innovation effects. Consequently, it further drives the transformation of industrial structures^35,36^). Based on the aforementioned information, this article posits an additional research hypothesis:

### H2

Digital finance fosters industrial restructuring, thereby fortifying the resilience of urban development.

During the phase of advanced economic development, it becomes imperative for economic growth to transition from being dependent on the input of production elements to enhancing the efficiency of those factors. The enhancement of production factors' efficiency is intrinsically linked to technological innovation or progress. This aligns with the perspective of endogenous economic growth theory, which posits that economic growth is contingent upon the economic system itself, with technological innovation serving as the wellspring for such growth. The demographic dividend has been impacted by the aging population, resulting in increased labor costs and decreased marginal efficiency of capital. However, it is crucial to note that the demographic dividend is influenced more by the quantity of talented individuals than the overall population size. Furthermore, the emergence of a talent dividend has markedly heightened the activity of innovation and entrepreneurship. Promoting widespread innovation and entrepreneurship has the potential to invigorate the enthusiasm of talented individuals in the field of innovation and entrepreneurship. This, in turn, can enhance the overall productivity of cities by optimizing the allocation of resources. Additionally, it enables the advancement of industrial foundations, the modernization of industrial chains, and the optimization of industrial structures, leading to improvements in quality and efficiency. This, in turn, cultivates new growth drivers exemplified by emerging industries, innovative formats, and novel models. Furthermore, the enhancement of urban innovation capabilities has had a notable impact on the quality of the workforce, leading to a greater degree of matching between industries and labor. This has resulted in the creation of employment prospects for surplus labor and mitigated the volatility of labor demand following a shock to a specific industry within the urban setting. It can be seen that innovation and entrepreneurship can establish new economic development spots and encourage the upward trend of urban resilience.

The advancement of urban innovation and entrepreneurship encounters two primary hurdles, namely financing difficulties and information asymmetry, due to the constraints of the conventional financial system. These challenges contribute to elevated levels of risk and uncertainty in innovation and entrepreneurship endeavors. The organic integration of digital technology and traditional finance has resulted in a new financial format known as digital inclusive finance. This format has effectively addressed the issues of imbalanced and inadequate development in traditional finance. Additionally, it has reduced the barriers to accessing financial services, enhanced financial accessibility for marginalized populations, and successfully mitigated the challenges of limited financing opportunities and information asymmetry encountered by innovators and entrepreneurs. Digital finance, in contrast to traditional financial lending practices, eliminates the need for collateral by leveraging digital technology to gather information on borrowers' account activity, consumption level, and risk preferences. This data is then utilized to assess creditworthiness, thereby lowering the barriers to accessing financial services and reducing the costs associated with lending. Consequently, digital finance facilitates the provision of credit to small and medium-sized enterprises as well as vulnerable populations, alleviating constraints related to financing and fostering entrepreneurial endeavors for both individuals and businesses. Furthermore, the presence of information asymmetry serves as a significant factor contributing to the strain between financial institutions and businesses, exacerbating the challenges faced by small and medium-sized organizations in obtaining financing^37–39^. The rapidly vigorous advancement of digital finance has resulted in the phenomenon of creative destruction inside the realm of traditional finance, necessitating traditional financial institutions to undertake a process of digital transformation. The establishment of a digital financial platform facilitates direct information exchange between parties seeking funds, resulting in reduced costs and time associated with information searches. This enables efficient matching of information across various entities, mitigating the extent of information asymmetry between banks and enterprises. Consequently, it helps to prevent adverse selection and moral hazard issues within the financial market. By expanding the range of financing methods and channels available to microentities requiring capital, there will be a concurrent increase in opportunities for both firms and individuals, thereby fostering innovation and entrepreneurship. Hence, the third hypothesis stated in this scholarly study is presented:

H3: Digital finance is conducive to invigorating the vitality of innovation and entrepreneurship, providing essential support for the development of resilient cities.

## Research design

### Model setting

Based on a theoretical analysis and research hypotheses, and building on the approach proposed by^40^, we have developed a fixed-effect model, denoted as Eq. (1), to examine the influence of digital finance on the resilience of cities.

1 $${\text{Re}} s_{it} = \alpha_{0} + \alpha_{1} difi_{it} + \alpha_{2} control_{it} + \mu_{i} + v_{t} + \varepsilon_{it}$$

In this context, ${\text{Res}}_{\text{it}}$ denotes the resilience level of city *i* at time* t*, whereas ${\text{difi}}_{\text{it}}$ represents the digital financial development level of city *i* at time *t*. The term ${\text{control}}_{\text{it}}$ refers to a collection of urban-level control variables. The utilization of individual fixed effects serves to control the impact of elements at the city level that remain constant over time, whereas the year fixed effect is employed to control variations in resilience over different time periods within the same city. The term $\varepsilon_{it}$ represents a stochastic error term, commonly referred to as a random disturbance term.

### Description of the data

#### Dependent variable

The primary independent variable examined in this scholarly paper is urban resilience (res), which pertains to the capacity of urban areas to withstand and recover from various shocks, maintain sustainability, and facilitate economic development. Drawing from the research methodology outlined by Li et al.^41^, we have chosen 15 indicators across three dimensions: resilience to risks (res1), adaptability and adjustment capacity (res2), and growth and recovery potential (res3) in order to establish an evaluation index system for urban resilience. Table 1 displays the precise indicators and their corresponding weights.

**Table 1:** Comprehensive evaluation index system for urban resilience.

First-Level Indicators	Second-level indicators	Indicator definitions	Weight
Resilience to risks	Economicstrength	Regional gross domestic product	0.1225
	Income level	Average wages of on-the-job employees	0.0134
	Foreign trade dependence	Import and export volume as a proportion of GDP	0.0018
	Employment pressure	Registered unemployed persons in cities and towns	0.0001
	Financial risk	Ratio of year-end loan balance to the deposit balance of the financial institution's	0.0002
Adaptability and adjustment ability	Investment scale	Investment in fixed assets	0.0891
	Consumption ability	Total retail sales of consumer goods	0.1197
	Fiscal self-sufficiency rate	Fiscal revenue and expenditure ratio	0.0317
	Level of savings	Savings of urban and rural residents	0.1247
	Social security	Number of beds in hospitals and health centers	0.0625
Growth and recovery ability	Technology investment	The proportion of science and technology expenditure in fiscal expenditure	0.0831
	Educational input	Education expenditure accounts for a proportion of fiscal expenditure	0.0083
	Innovation output	Number of patents granted	0.2498
	Human capital	Number of students enrolled in institutions of higher learning	0.1801
	Industrial structure	Ratio of the added value of the tertiary industry to the secondary industry	0.0324

#### Independent variable

The core explanatory variable in this article is digital finance (difi). The Digital Inclusive Finance Index is employed as a pr» indicator in accordance with the methodology proposed by Guo et al.^42^. The Digital Inclusive Finance Index was created by the Peking University Digital Finance Research Center, utilizing extensive data provided by Ant Financial. This index incorporates 33 distinct indicators derived from three fundamental aspects, namely coverage breadth, depth of use, and degree of digitalization, to assess the state of digital inclusive finance. It precisely and objectively assesses the comprehensive development level of digital finance at provincial, city, and district levels in China. This index serves as a quantitative reference tool for research related to digital finance. To mitigate the impact of outliers on the regression outcomes, this study employs logarithmic processing on the chosen “Peking University Digital Inclusive Finance Index (Phase III, 2011–2020)”.

#### Control variables

In order to comprehensively assess the influence of digital finance on urban resilience and draw from established research practices^43^^,^^44^, this study incorporates variables such as population density, economic development level, marketization level, and informatization leve as control variables. Population density, denoted as “pop,” is a metric that quantifies the number of individuals residing within a certain area, typically measured in terms of the number of people per square kilometer. The economic development level, also known as per capita GDP (PGDP), is quantified by taking the logarithm of the per capita GDP for each city. The marketization level, denoted as “market,” is measured by the ratio of private and individual employees in urban areas to the total number of employees in urban units at the end of a certain period. This ratio serves as a proxy variable for assessing the extent of marketization. The informatization level (infma) is a metric utilized to assess the degree of informatization within a certain region. This measurement is determined by considering the number of mobile phones per capita present within the territory.

The data utilized for the computation of urban resilience and urban characteristic variables is sourced from the China Urban Statistical Yearbook, China Statistical Yearbook, statistical bulletins of each city for the corresponding year, CSMAR, and the Wind database. During the course of actual data processing, we conducted a matching procedure between digital financial indicators and urban data. Subsequently, we performed data cleaning procedures, resulting in the creation of a panel dataset comprising 287 cities at the prefecture level and above in China. The dataset covers the time period from 2011 to 2020. Table 2 displays the descriptive statistics for the primary factors examined in this study.

**Table 2:** Descriptive statistics of the variables.

Variables	Obs.	Mean	Std. dev.	Min.	Max.
*res*	2870	0.0878	0.0872	0.0186	0.7965
*res1*	2870	0.0138	0.0124	0.0026	0.1367
*res2*	2870	0.0360	0.0346	0.0021	0.3258
*res3*	2870	0.0380	0.0430	0.0054	0.4025
*difi*	2870	5.0542	0.5162	2.8344	5.8126
*cov*	2870	4.9834	0.5750	0.6206	5.7884
*dep*	2870	5.0342	0.5175	1.4563	5.8572
*dig*	2870	5.2181	0.6132	0.9933	6.3651
*pop* *pgdp* *market*	2870 2870 2870	5.7339 10.7154 1.2560	0.9304 0.5739 0.8403	1.6094 8.7729 0.0152	7.8816 13.0557 17.1414
*infma*	2870	0.6233	2.5161	0.0001	39.5800

### Temporal evolution characteristics

#### Demonstration of the core density of urban resilience

**Figure 1:**
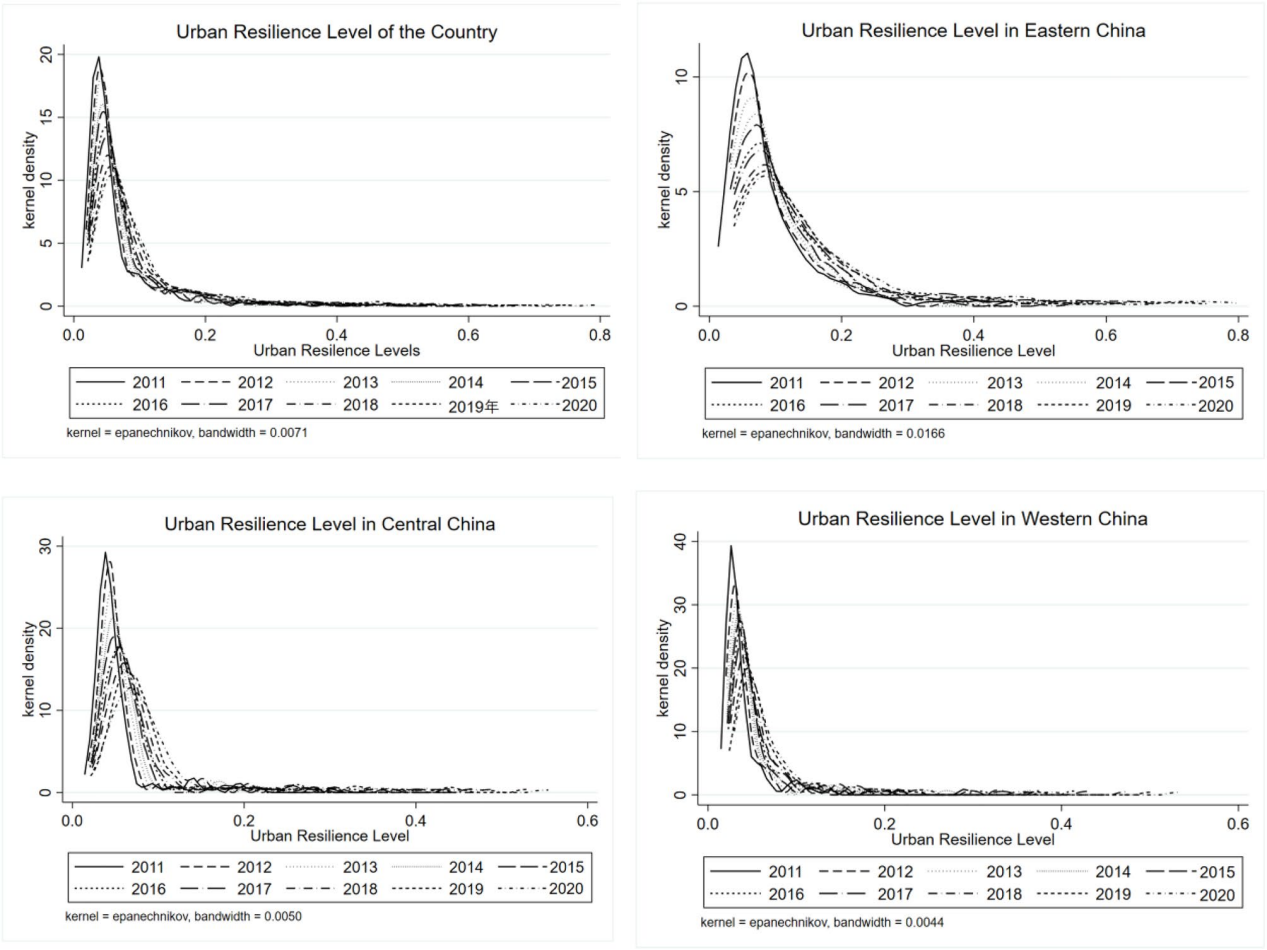
Dynamic distribution of urban resilence levels in China and various regions from 2011 to 2020.

Figure 1 depicts the dynamic kernel density distribution of urban resilience levels across different regions in China. Upon examination of the distribution curves depicting the resilience levels of cities at the national and regional scales, several shared patterns become apparent. During the sample period, it can be observed from the kernel curve that the primary peak of the urban resilience level in both countries and regions has shifted towards higher values. This shift suggests a consistent improvement in the urban resilience level across all areas. In relation to the extent of displacement, the eastern region exhibits the greatest degree, followed by the middle region, while the western region demonstrates the lowest degree. This suggests the presence of regional disparities in the degree of resilience. In terms of peak shape, it can be observed that the western region exhibits the greatest peak height, followed by the eastern region, while the eastern region displays the lowest peak height. This suggests that there is minimal disparity in urban resilience levels between the central and western regions. From the standpoint of distribution morphology, a phenomenon of tailing is observed in all regions, with the right tail progressively elongating over the years. This suggests a widening trend in the extensibility of the distribution, indicating a gradual widening of the spatial disparity in urban resilience levels across the nation.

#### Basic characteristics of digital finance

**Figure 2:**
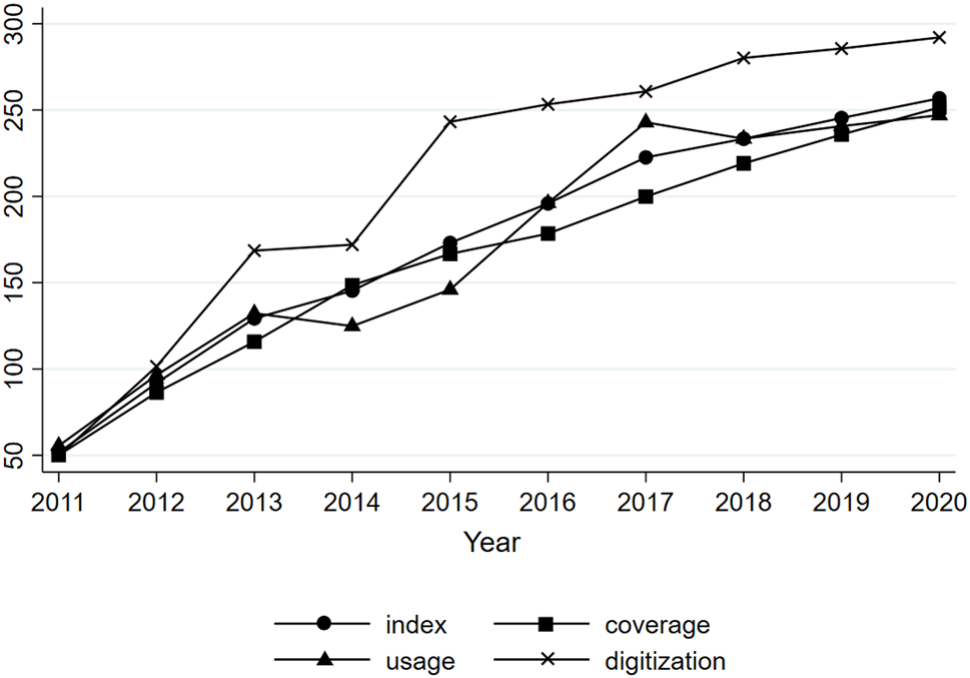
Changes in the level of urban digital finance development from 2011 to 2020.

Figure 2 represents the changes in the development level of digital finance and its three different dimensions in Chinese cities at the prefecture level and above from 2011 to 2020. The picture illustrates the upward trend in the level of digital finance in urban areas, albeit with varying growth rates across different years. The period between 2011 and 2013 had the most substantial growth rate, particularly in 2012, when it reached an impressive 77.65%. Between the years 2014 and 2017, the growth rate showed fluctuations within the range of 10% to 20%. Following the year 2018, there was a resurgence in the growth rate, which subsequently exhibited fluctuations hovering around the 5% mark. China’s urban digital finance has exhibited significant advancements. The scope, depth of utilization, and level of digitalization have exhibited a changing and rising trajectory from many perspectives. Of these, the dynamic trend of coverage breadth closely aligns with the overall level of the Digital Finance Index, underscoring the significance of coverage breadth in enhancing the level of digital finance in Chinese cities. The rate of digitalization had a rapid rise during the years 2011 and 2015, followed by a subsequent deceleration in growth.

**Figure 3:**
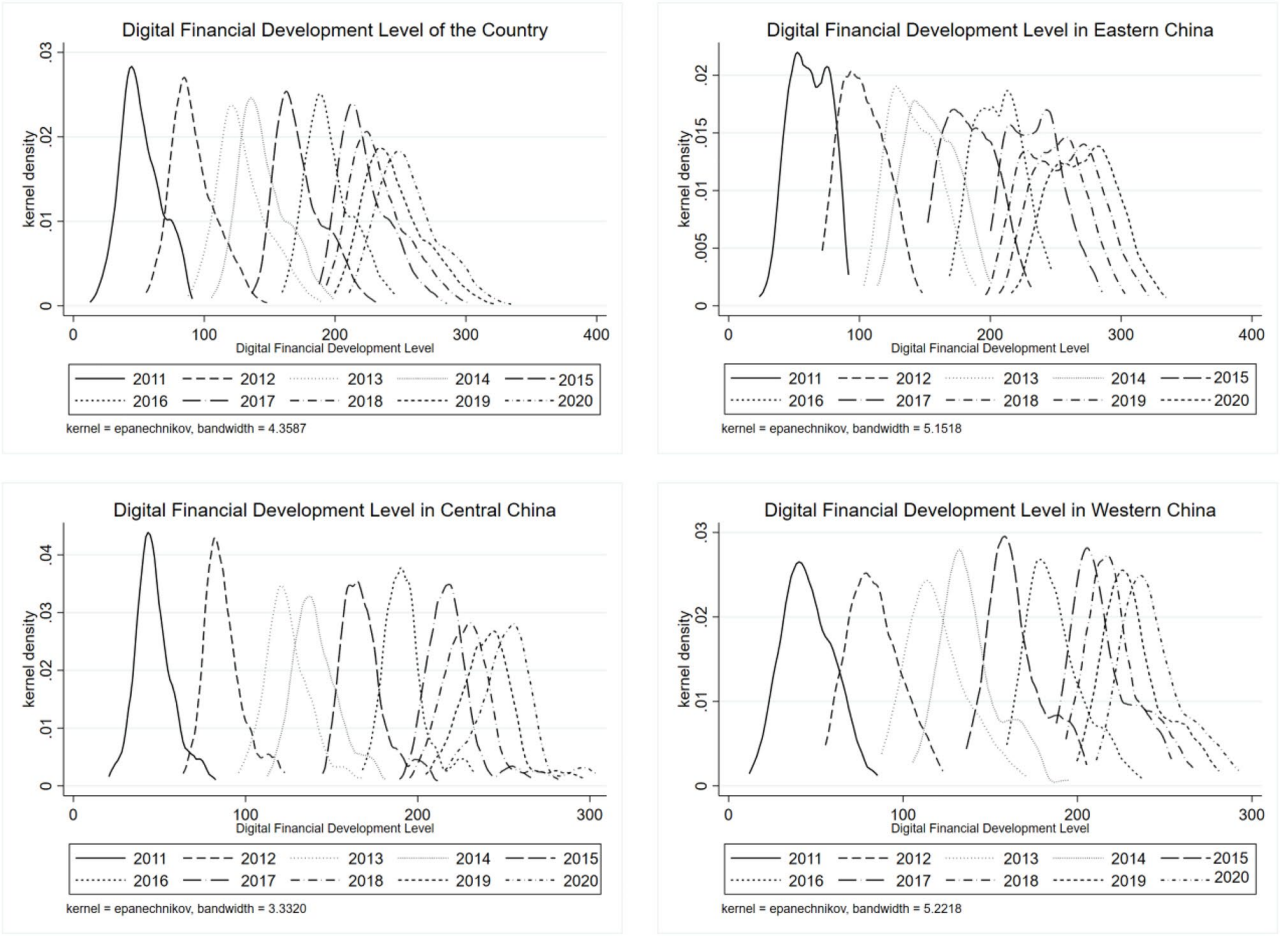
Dynamic distribution of the development level of digital finance in cities across the country and regions from 2011 to 2020.

Figure 3 illustrates the kernel density plot of the digital financial index in China and its three regions (East, Central, and West) from 2011 to 2020. This figure aims to highlight the variations in the level of regional digital financial development. From a peak movement standpoint, the primary peak position of the distribution curve representing the developmental level of digital inclusive finance in Chinese cities at or above the prefecture level, as well as the three major regions of East, Central, and West, has experienced a notable rightward shift from 2011 to 2020. This shift suggests that the developmental level of digital finance in each region has undergone leapfrog advancements, surpassing previous levels. Additionally, it indicates a gradual decline in the developmental level of digital finance across the East, Central, and Western regions. From a distribution morphology standpoint, it can be observed that the nuclear density distribution curve exhibits a declining tendency in the eastern and central regions, while the western region displays a pattern characterized by a decline followed by an increase and then another decline. The observed phenomenon of the digital financial kernel density distribution curve widening across different regions suggests an increasing disparity and inadequacy in the development of digital finance. This is accompanied by a growing internal divergence and a spatial imbalance. Furthermore, the eastern region exhibits a weak “bimodal” characteristic, while other regions display a distinct “unimodal” distribution. This indicates the absence of a multipolarization phenomenon in digital inclusive finance among regions. The digital finance index of prefecture-level cities in the central region exhibits a right-tailing phenomenon when considering distribution and scalability. This observation suggests that there are notable variations in the advancement of digital finance among cities in the central region. In contrast, the eastern and western regions do not display a pronounced right-tailing phenomenon.

## Empirical results and analysis

### Basic regression analysis

Table 3 reports the benchmark regression results for the impact of digital finance on urban resilience. In Column 1, the regression analysis is conducted just on the core explanatory variable. However, in Columns (2–5), additional control factors are introduced in a sequential manner. These control variables include population density, economic development level, marketization level, and transportation convenience. It is evident that, irrespective of the control of other factors, the coefficient of digital finance demonstrates statistical significance at the 1% level, with a positive sign. The empirical findings indicate that digital finance plays a significant role in fostering urban resilience, hence boosting cities’ capacity to withstand risk shocks, sustainabilitiy, and recover from growth setbacks. The regression results for digital finance on urban resilience sub-items are also displayed in columns (6–8), indicating notable variations in impact. The empirical findings indicate that the regression coefficients associated with digital finance exhibit a statistically significant positive relationship at a confidence level of 1%. This relationship holds true irrespective of risk aversion, adaptability, or growth recuperation. In contrast, it can be observed that the growth recovery coefficient exhibits the highest value, while the risk resistance coefficient displays the lowest value. These findings reflect the focus of digital finance on supporting resilient cities. Digital finance has the more potential to enhance cities’ capacity to adapt, adjust, and recover from growth. This is primarily achieved through the provision of financial assistance, the alleviation of financing constraints and credit pressures on the demand side, the stimulation of market economy adjustments, and the resumption of normal operations. The level of support provided by digital finance in mitigating risks for urban areas is comparatively low, as indicated by the research conducted by^45^ (Zhou et al. 2022). This observation, to some degree, reflects the current state of digital finance in China and its effectiveness in managing risks within the economic domain. In forthcoming advancements, it is imperative to prioritize the path of reform aimed at preventing and resolving significant regional financial risks while concurrently attaining steady and sound economic growth. This should be done in conjunction with the distinctive digital information attributes of the digital financial model.

**Table 3:** Basic regression model results.

Variables	*res*	*res*	*res*	*res*	*res*	*res1*	*res2*	*res3*
	(1)	(2)	(3)	(4)	(5)	(6)	(7)	(8)
*difi*	0.0251 ***	0.0228***	0.0130***	0.0123***	0.0123***	0.0021***	0.0046***	0.0055***
	(30.46)	(28.00)	(11.10)	(10.33)	(10.28)	(10.73)	(9.82)	(8.72)
*pop*		0.1796***	0.1649***	0.1653***	0.1661***	0.0225***	0.0471***	0.0965***
		(13.47)	(12.60)	(12.68)	(12.54)	(10.57)	(8.95)	(13.64)
*pgdp*			0.0293***	0.0282***	0.0282***	0.0066***	0.0146***	0.0070***
			(11.25)	(10.81)	(10.81)	(15.75)	(14.11)	(5.01)
*market*				0.0029***	0.0029***	0.0005***	0.0011***	0.0013***
				(4.37)	(4.36)	(4.95)	(4.13)	(3.60)
*infma*					0.0001	0.0001	− 0.0001	0.0001
					(0.34)	(1.27)	(− 0.43)	(0.58)
*_cons*	− 0.3902***	− 1.0572***	− 1.2374***	− 1.2280***	− 1.2327***	− 0.1973***	− 0.4158***	− 0.6195***
	(− 9.33)	(− 13.96)	(− 16.36)	(− 16.28)	(− 16.08)	(− 16.01)	(− 13.66)	(− 15.14)
City fixed	YES	YES	YES	YES	YES	YES	YES	YES
Year fixed	YES	YES	YES	YES	YES	YES	YES	YES
Obs	2870	2870	2870	2870	2870	2870	2870	2870
${R}^{2}$	0.2644	0.3127	0.3448	0.3496	0.3496	0.4273	0.3732	0.2351
F	133.80	116.21	93.35	93.73	93.09	70.81	91.72	86.69
*P*	0.0000	0.0000	0.0000	0.0000	0.0000	0.0000	0.0000	0.0000

*, **, and ***indicate passing the significance test at 10%, 5%, and 1% levels, respectively.

### Robustness checks

#### Endogenous analysis

The benchmark regression analysis assessed the influence of digital finance on urban resilience and its many sub-components. The findings indicated a significant impact; nevertheless, it is important to consider potential regression bias arising from reverse causality and omitted factors. Consequently, this essay employs two methodologies to address endogenous concerns: The utilization and advancement of digital finance can be influenced by the degree of urban resilience, encompassing several aspects such as consumption, investment, production, and other related activities. In order to mitigate the potential bias of reverse causality, this work proceeds to reassess the digital finance index by introducing a lag of one order. Furthermore, it is important to acknowledge that despite the comprehensive inclusion of various control variables, there are certain factors, such as government behavior and cultural customs, which are challenging to acquire and cannot be precisely quantified. These factors can also exert an influence on economic development. Additionally, it is crucial to recognize that the endogenous nature of relevant variables cannot be disregarded. Thus, building upon the methodology proposed by Fu and Huang^46^, we employed the distance from each prefecture-level city to Hangzhou as an instrumental variable in order to substantiate our hypothesis. This article primarily utilizes the latitude and longitude coordinates of each city and Hangzhou in order to compute the geographical distance between them. This calculation is based on the formula for determining the distance between two locations on a sphere. The fixity of geographical distance is unaffected by external causes due to its typical natural geographical properties. Furthermore, there is no direct association shown between geographical distance and urban resilience, hence satisfying the stringent exogenous criteria required for instrumental variables. When the lagging first order of digital finance is used as an instrumental variable, the regression coefficient is positive, indicating that the development level of digital finance is generally increasing. When the distance to Hangzhou is used as an instrumental variable, the regression coefficient is negative, indicating that the level of digital finance development in Chinese cities is negatively related to the distance from Hangzhou to Hangzhou. Both are in line with China's actual development situation. Based on the findings presented in Table 4, it is evident that the regression outcomes for the digital finance index lagging one step and instrumental variables have successfully passed the tests for “unidentifiable” and “weak instrumental variables”. These results suggest that the instrumental variables chosen in this study are both effective and justifiable. The regression coefficients pertaining to digital finance and its impact on urban resilience and its sub-capabilities exhibit a statistically significant positive relationship at a significance level of 1%. In addition to providing further evidence of the beneficial impact of digital money on urban resilience, this also serves to validate the accuracy and dependability of the benchmark findings.

**Table 4:** Robustness test: instrumental variable regression.

	*res1*		*res2*		*res3*	
	L.difi	distance	L.difi	distance	L.difi	distance
	(1)	(2)	(3)	(4)	(5)	(6)
*difi*	0.0157***	0.0609***	0.0287***	0.1847***	0.0336***	0.2104***
	(16.99)	(5.33)	(15.78)	(5.27)	(14.46)	(5.18)
*_cons*	− 0.0457***	− 0.2942***	− 0.1118***	− 0.8978***	− 0.1353***	− 1.0255***
	(− 13.57)	(− 5.09)	(− 12.38)	(− 5.07)	(− 11.70)	(− 5.00)
Controls	YES	YES	YES	YES	YES	YES
City fixed	YES	YES	YES	YES	YES	YES
Year fixed	YES	YES	YES	YES	YES	YES
Underidentification test	760.820 [0.0000]	25.487 [0.0000]	760.820 [0.0000]	25.487 [0.0000]	760.820 [0.0000]	25.487 [0.0000]
Weak variable test	3.7e+04 {16.38}	25.698 {16.38}	3.7e+04 {16.38}	25.698 {16.38}	3.7e+04 {16.38}	25.698 {16.38}
Obs	2583	2870	2583	2870	2583	2870

*, **, and *** indicate passing the significance test at 10%, 5%, and 1% levels, respectively.

#### Replacement of the core explanatory variable processing method

In the benchmark regression analysis, the digital financial index was employed as the independent variable for regression analysis. However, it is important to acknowledge the potential presence of regression bias. We replace the treatment method of digital finance indicators to conduct a robustness test. The regression results pertaining to the influence of digital finance on resilience, adaptation, adjustment, and innovation transformation in resilient cities are presented in Table 5, subsequent to the replacement of the treatment technique. It is evident that despite a drop in the regression coefficient value and significance of digital finance, it continues to play a positive role in fostering urban resilience and its sub-indicators. This observation partially confirms the reliability and stability of the benchmark regression outcomes.

**Table 5:** Robustness test: replacement of the core explanatory variable processing method.

	*res*	*res*	*res1*	*res2*	*res3*
	(1)	(2)	(3)	(4)	(5)
*difi*	0.0230***	0.0183**	0.0026**	0.0059***	0.0077**
	(3.85)	(2.24)	(2.32)	(2.93)	(2.34)
*_cons*	0.0477***	− 0.8994***	− 0.0988***	− 0.2943***	− 0.3533***
	(44.39)	(− 12.16)	(− 11.78)	(− 12.00)	(− 10.91)
Controls	No	Yes	Yes	Yes	Yes
City fixed	Yes	Yes	Yes	Yes	Yes
Year fixed	Yes	Yes	Yes	Yes	Yes
Obs	2870	2870	2870	2870	2870
${R}^{2}$	0.3809	0.4194	0.6181	0.4851	0.3425
F	153.80	103.89	114.24	114.46	127.93
*P*	0.0000	0.0000	0.0000	0.0000	0.0000

*, **, and ***indicate passing the significance test at 10%, 5%, and 1% levels, respectively.

#### Adjusting the sample range

The temporal coverage of the digital financial variables in the entire sample ranges from 2011 to 2020, commencing prior to the commonly acknowledged inception year of digital finance, which is 2013. To mitigate potential biases in the temporal selection, this paper excludes data from the years 2011 and 2012 and subsequently re-estimates the model by utilizing samples from the remaining years. The new findings are presented in Table 6, whereby columns (1) and (2) employ urban resilience as the independent variable, while columns (3–5) employ sub-indicators of urban resilience as the independent variable. The results indicate that when adjusting the sample size, the regression coefficients for digital finance in each model have exhibited a drop. However, it is noteworthy that all coefficients have still passed the significance test at the 1% level. This finding supports the research conclusions derived from the benchmark regression results, hence highlighting the robustness of the findings.

**Table 6:** Robustness test: adjusting the sample range.

	*res*	*res*	*res1*	*res2*	*res3*
	(1)	(2)	(3)	(4)	(5)
*difi*	0.0476***	0.0364***	0.0050***	0.0100***	0.0172***
	(28.79)	(15.11)	(19.16)	(13.17)	(16.02)
*_cons*	− 0.1580***	− 1.2365***	− 0.1236***	− 0.3933***	− 0.4879***
	(− 18.13)	(− 13.72)	(− 12.56)	(− 13.77)	(− 12.15)
Controls	No	Yes	Yes	Yes	Yes
City fixed	Yes	Yes	Yes	Yes	Yes
Year fixed	Yes	Yes	Yes	Yes	Yes
Obs	2296	2296	2296	2296	2296
${R}^{2}$	0.2922	0.3464	0.5523	0.3877	0.3020
F	153.67	107.39	125.05	128.12	121.73
*P*	0.0000	0.0000	0.0000	0.0000	0.0000

*, **, and ***indicate passing the significance test at 10%, 5%, and 1% levels, respectively.

#### Censored treatment

In order to mitigate the effects of macroeconomic fluctuations in cities at the prefecture level and higher, as well as extreme values in the collected data, we conducted bilateral trimming on the panel data at the 5% quantile. This involved replacing values that exceeded the 95% quantile with the value at the 95% quantile, and replacing values that fell below the 5% quantile with the value at the 5% quantile. Subsequently, the reduced samples were subjected to regression analysis once more. The regression analysis are presented in Table 7, conducted after removing extreme values, reveals that digital finance has a statistically significant and beneficial impact on urban resilience and its sub-indicators. These findings are similar to the conclusions obtained from the benchmark regression analysis.

**Table 7:** Robustness test: censored treatment.

	*res*	*res*	*res1*	*res2*	*res3*
	(1)	(2)	(3)	(4)	(5)
*difi*	0.0240***	0.0113***	0.0018***	0.0045***	0.0049***
	(34.92)	(11.22)	(13.03)	(11.09)	(9.13)
*_cons*	− 0.0348***	− 0.7267***	− 0.1199***	− 0.3159***	− 0.2980***
	(− 9.94)	(− 10.68)	(− 12.57)	(− 11.63)	(− 8.17)
Controls	No	Yes	Yes	Yes	Yes
City fixed	Yes	Yes	Yes	Yes	Yes
Year fixed	Yes	Yes	Yes	Yes	Yes
Obs	2870	2870	2870	2870	2870
${R}^{2}$	0.3207	0.3947	0.5508	0.4259	0.2426
F	172.93	115.21	97.84	102.99	110.89
*P*	0.0000	0.0000	0.0000	0.0000	0.0000

*, **, and ***indicate passing the significance test at 10%, 5%, and 1% levels, respectively.

#### Excluding key cities

Due to policy bias, certain regional units, such as municipalities under the central government, cities designated in the state plan, and provincial capitals, may receive disproportionate financial resources and fiscal and tax support compared to ordinary prefecture-level cities. As a result, these regions may exhibit macroeconomic data that deviates from the expected range. It is imperative to put up a proposal and thereafter re-validate the robustness of the regression outcomes. In accordance with the methodology employed by Wang et al.^27^, this study excluded the aforementioned major cities from subsequent analysis in order to assess the influence of digital finance on urban resilience. Table 8 presents data on various municipalities in China. Columns (1–3) do not include four municipalities that fall under the jurisdiction of the central government, as well as 27 province capitals. Similarly, columns (4–6) exclude five cities that have been officially designated in the state plan. Lastly, columns (7–9) exclude 15 sub-provincial cities. The findings indicate that the regression coefficient of digital finance on urban resilience remains significantly positive, regardless of the exclusion of municipalities directly under the central government or other types of key cities. This demonstrates a clear promoting effect and reinforces the robustness of the research conclusion presented in this article.

**Table 8:** Robustness test: excluding key cities.

	*res1*	*res2*	*res3*	*res1*	*res2*	*res3*	*res1*	*res2*	*res3*
	(1)	(2)	(3)	(4)	(5)	(6)	(7)	(8)	(9)
*difi*	0.0016***	0.0032***	0.0036***	0.0018***	0.0042**	0.0049**	0.0016***	0.0038***	0.0041***
	(14.12)	(11.09)	(7.48)	(9.83)	(9.12)	(8.32)	(10.06)	(8.95)	(8.17)
*_cons*	− 0.1721***	− 0.3374***	− 0.6240***	− 0.1309***	− 0.3394***	− 0.3422***	− 0.0950***	− 0.2542***	− 0.2407***
	(− 22.06)	(− 16.86)	(− 18.66)	(− 10.70)	(− 10.76)	(− 8.65)	(− 8.66)	(− 8.99)	(− 7.06)
Controls	Yes	Yes	Yes	Yes	Yes	Yes	Yes	Yes	Yes
City fixed	Yes	Yes	Yes	Yes	Yes	Yes	Yes	Yes	Yes
Year fixed	Yes	Yes	Yes	Yes	Yes	Yes	Yes	Yes	Yes
Obs	2560	2560	2560	2820	2820	2820	2720	2720	2720
${R}^{2}$	0.6155	0.5099	0.2731	0.4384	0.3763	0.2174	0.4617	0.3833	0.2262
F	72.25	69.86	34.79	75.31	97.64	102.69	84.34	103.01	86.87
*P*	0.0000	0.0000	0.0000	0.0000	0.0000	0.0000	0.0000	0.0000	0.0000

*, **, and ***indicate passing the significance test at 10%, 5%, and 1% levels, respectively.

## 5 Further analysis

### Heterogeneity analysis

#### Heterogeneity of digital financial structures

Given that the digital finance index encompasses various characteristics, such as the breadth of coverage, depth of usage, and degree of digitalization, it becomes imperative to conduct a more comprehensive examination of the structural implications of digital finance on economic resilience. The regression results for the three separate capabilities encompassed in economic resilience, specifically the coverage breadth, depth of use, and degree of digitalization, of digital finance, are presented in Table 9. Columns (1), (4), and (7) display the results for the first capability; columns (2), (5), and (8) present the results for the second capability; and columns (3), (6), and (9) showcase the results for the third capability. The table illustrates that the three dimensions of digital finance have played a crucial role in enhancing the resilience of cities. In terms of their influence, the depth of utilization of digital finance exhibits the highest impact, followed by the degree of digitalization, while the coverage dimension demonstrates the lowest impact. This observation also suggests that there is a need to persistently prioritize and broaden the scope of future digital financial advancements. This would enable those residing in rural places and marginalized communities inside urban areas to access a wider selection of high-quality and efficient financial services. Furthermore, it is worth noting that the three aspects of digital finance have a greater impact on cities' capacity to adapt, change, and innovate, compared to their ability to mitigate risks. There are several potential factors that could contribute to this phenomenon. With the increasing prevalence of digital finance within diverse socioeconomic cohorts, individuals now have enhanced access to a range of digital financial services, including but not limited to mobile payments, credit facilities, insurance coverage, and investment opportunities. The establishment of a lower threshold in financial services presents increased prospects for economically disadvantaged people to generate wealth. Furthermore, the development of digital infrastructure has significantly enhanced the degree of social information, facilitated the restructuring of industries, maximized the allocation of financial and other resources, and served as a catalyst for the continued advancement of the economy and society. Moreover, it is important to note that digital finance remains inherently financial in nature, and as such, it is not exempt from the associated hazards that come with traditional finance. Despite the utilization of digital technologies such as blockchain and artificial intelligence, which offer technological assistance in mitigating and addressing substantial risks, their impact remains insufficient to substantially alter the limitations imposed by the city's inherent economic structure in order to effectively withstand external disruptions.

**Table 9:** Regression results of heterogeneity in digital financial structure.

	*res1*			*res2*			*res3*		
	(1)	(2)	(3)	(4)	(5)	(6)	(7)	(8)	(9)
*cov*	0.0012***			0.0028***			0.0035***		
	(6.58)			(6.51)			(5.97)		
*dep*		0.0022***			0.0044***			0.0060***	
		(12.02)			(9.68)			(9.87)	
*dig*			0.0019***			0.0043***			0.0045***
			(13.53)			(12.68)			(9.70)
*_cons*	− 0.2099***	− 0.1981***	− 0.1975***	− 0.4406***	− 0.4264***	− 0.4144***	− 0.6471***	− 0.6207***	− 0.6303***
	(− 16.78)	(− 16.29)	(− 16.43)	(− 14.30)	(− 14.11)	(− 13.95)	(− 15.68)	(− 15.35)	(− 15.65)
*Controls*	Yes	Yes	Yes	Yes	Yes	Yes	Yes	Yes	Yes
City fixed	Yes	Yes	Yes	Yes	Yes	Yes	Yes	Yes	Yes
Year fixed	Yes	Yes	Yes	Yes	Yes	Yes	Yes	Yes	Yes
Obs	2870	2870	2870	2870	2870	2870	2870	2870	2870
${R}^{2}$	0.4116	0.4335	0.4414	0.3603	0.3726	0.3880	0.2233	0.2412	0.2403
F	68.91	71.62	72.89	89.76	91.67	94.31	85.20	87.53	87.47
*P*	0.0000	0.0000	0.0000	0.0000	0.0000	0.0000	0.0000	0.0000	0.0000

*, **, and ***indicate passing the significance test at 10%, 5%, and 1% levels, respectively.

#### Location heterogeneity

The extant body of literature has demonstrated that digital finance exhibits notable variations in its influence on economic resilience across diverse geographies. In China, the cities that are classified as prefecture-level and higher are categorized into three distinct economic areas, namely East, Central, and West. The analysis delves deeper into the geographical variations in the influence of digital finance on urban resilience. The findings are presented in Table 10. The findings of regression analysis for cities in the eastern, central, and western regions are presented in columns (1), (4), (7), (2), (5), (8), and (3), (6), (9), respectively. Based on the findings of the regression analysis, it is evident that digital finance exerts a statistically significant and beneficial impact on urban resilience among China's three primary economic regions, namely the East, Central, and West. Also, this positive effect remains consistent across different regional characteristics. Furthermore, it is seen that digital finance has a notably greater impact on promoting urban resilience in the eastern region compared to the central and western regions. This discrepancy may be attributed to the eastern region's superior economic development and more balanced industrial structure. Finally, it is observed that the impact of digital finance on the flexibility and innovative transformation capabilities of the three key areas is considerably greater compared to its capacity to mitigate risks.

**Table 10:** Regional heterogeneity of digital finance's impact on urban resilience.

	*Res1*			*Res2*			*Res3*		
	eastern	central	western	eastern	central	western	eastern	central	western
	(1)	(2)	(3)	(4)	(5)	(6)	(7)	(8)	(9)
*difi*	0.0032***	0.0011***	0.0012***	0.0074***	0.0028***	0.0027***	0.0075***	0.0027***	0.0026***
	(7.09)	(5.20)	(6.35)	(7.35)	(4.10)	(4.49)	(5.14)	(3.76)	(3.13)
*_cons*	− 0.3599***	− 0.0495***	− 0.0977***	− 0.6576***	− 0.1399**	− 0.2696***	− 1.3270***	0.0563	− 0.3377***
	(− 11.86)	(− 2.76)	(− 9.81)	(− 9.83)	(− 2.47)	(− 8.89)	(− 13.71)	(0.94)	(− 8.04)
*controls*	Yes	Yes	Yes	Yes	Yes	Yes	Yes	Yes	Yes
City fixed	Yes	Yes	Yes	Yes	Yes	Yes	Yes	Yes	Yes
Year fixed	Yes	Yes	Yes	Yes	Yes	Yes	Yes	Yes	Yes
Obs	2870	2870	2870	2870	2870	2870	2870	2870	2870
${R}^{2}$	0.4555	0.5452	0.5876	0.4292	0.4467	0.3748	0.3323	0.3070	0.2359
F	66.09	77.30	51.74	92.15	97.38	85.75	63.45	154.05	129.47
*P*	0.0000	0.0000	0.0000	0.0000	0.0000	0.0000	0.0000	0.0000	0.0000

*, **, and ***indicate passing the significance test at 10%, 5%, and 1% levels, respectively.

#### Intermediary effect test

In the preceding section, we have illustrated the extent to which digital financial development has a direct impact on urban resilience, using benchmark regression analysis and robustness testing. However, additional investigation is required in order to comprehend the impact of digital finance on urban resilience. As previously examined in the part dedicated to theoretical analysis, the pivotal factors influencing urban resilience through the influence of digital finance are industrial transformation and innovation. Hence, this section examines the precise processes via which digital finance impacts urban resilience, focusing on the perspectives of industrial transformation and innovation. In this study, the authors Li and Shen^47^ propose the utilization of a composite index that combines measures of industrial rationalization and industrial modernization in order to assess and describe the process of industrial transformation. To assess urban innovation levels, we adopt the methodology, which involves utilizing the quantity of patent applications as a metric. The urban entrepreneurship level is determined by incorporating the proportion of urban private and self-employed workers in relation to the overall population. Given the premise that both innovation and entrepreneurship hold equal significance, each carrying a weight of 0.5, the subsequent step involves the computation of the degree of urban innovation and entrepreneurship.

If industrial transformation and innovation serve as the mechanisms through which digital finance influences urban resilience, it is imperative to initially investigate how digital finance impacts these two factors before delving into their impact on urban resilience. Thus, based on Model (1), this study tests whether digital finance influences urban resilience through its effects on industrial transformation and innovation, and subsequently formulates the following model:

2 $$M_{it} = \beta_{0} + \beta_{1} difi_{it} + \beta_{2} control_{it} + \mu_{i} + v_{t} + \varepsilon_{it}$$

3 $${\text{Re}} s_{it} = \gamma_{0} + \gamma_{1} difi_{it} + \gamma_{2} M_{it} + \gamma_{3} control_{it} + \mu_{i} + v_{t} + \varepsilon_{it}$$

In Eqs. (2) and (3), ${M}_{\text{it}}$ represents the level of industrial transformation and upgrading, as well as innovation and entrepreneurship, in city i during period t. The definitions of the other variables remain consistent with those in Eq. (1). Table 11 provides an account of the influence of digital finance on the levels of industrial transformation and upgrading, and innovation and entrepreneurship.

**Table 11:** The impact of digital finance on industrial structure transformation and innovation and entrepreneurship.

	*stru*				*inno*			
	(1)	(2)	(3)	(4)	(5)	(6)	(7)	(8)
*difi*	0.4361***				0.1498***			
	(24.21)				(13.73)			
*cov*		0.3851***				0.1264***		
		(23.51)				(12.75)		
*dep*			0.4248***				0.1412***	
			(24.53)				(13.38)	
*dig*				0.2230***				0.0814***
				(16.01)				(10.08)
*_cons*	1.0016	0.7063	− 0.0436	− 2.0109	0.6808	0.5685	0.2736	− 0.2441
	(0.85)	(0.59)	(− 0.04)	(− 1.63)	(0.99)	(0.82)	(0.40)	(− 0.35)
Ccontrols	Yes	Yes	Yes	Yes	Yes	Yes	Yes	Yes
City fixed	Yes	Yes	Yes	Yes	Yes	Yes	Yes	Yes
Year fixed	Yes	Yes	Yes	Yes	Yes	Yes	Yes	Yes
obs	2870	2870	2870	2870	2870	2870	2870	2870
${R}^{2}$	0.3352	0.3232	0.3385	0.2560	0.2122	0.2047	0.2095	0.1866
F	20.69	20.11	21.39	19.35	74.68	73.96	74.46	71.37
*P*	0.0000	0.0000	0.0000	0.0000	0.0000	0.0000	0.0000	0.0000

*, **, and ***indicate passing the significance test at 10%, 5%, and 1% levels, respectively.

Regarding the influence of digital finance on the process of industrial transformation and upgrading in prefecture-level cities, the findings presented in Table 12, specifically in column (1), demonstrate a statistically significant positive regression coefficient for digital finance at a significance level of 1%. Furthermore, columns (2–4) provide insights into the effects of the three dimensions of digital finance on industrial transformation and upgrading. The regression coefficients for these dimensions are also significantly positive at the 1% level, indicating that digital finance indeed plays a constructive role in promoting the advancement of urban industrial transformation and upgrading. The columns (5–8) in the table demonstrate the influence of digital finance and its three dimensions on innovation and entrepreneurship. The findings indicate that the primary explanatory factors exhibit statistically significant positive coefficients, suggesting that the advancement of digital finance has the potential to enhance innovation and entrepreneurship activities.

**Table 12:** Intermediate effect regression results.

	*res1*		*res2*		*res3*	
	(1)	(2)	(3)	(4)	(5)	(6)
*difi*	0.0013***	0.0015***	0.0031***	0.0033***	0.0028***	0.0035***
	(6.06)	(7.66)	(5.84)	(6.90)	(3.97)	(5.36)
*stru*	0.0011***		0.0022***		0.0048***	
	(5.21)		(4.20)		(6.80)	
*inno*		0.0023***		0.0058***		0.0106***
		(6.86)		(6.94)		(9.43)
*_cons*	− 0.2116***	− 0.1970***	− 0.4519***	− 0.4237***	− 0.6340***	− 0.6254***
	(− 16.77)	(− 16.53)	(− 14.45)	(− 14.36)	(− 15.28)	(− 15.84)
Controls	Yes	Yes	Yes	Yes	Yes	Yes
City fixed	Yes	Yes	Yes	Yes	Yes	Yes
Year fixed	Yes	Yes	Yes	Yes	Yes	Yes
obs	2870	2870	2870	2870	2870	2870
${R}^{2}$	0.4472	0.4481	0.3930	0.3932	0.2537	0.2655
F	62.65	58.95	80.61	71.91	70.78	67.02
*P*	0.0000	0.0000	0.0000	0.0000	0.0000	0.0000

*, **, and ***indicate passing the significance test at 10%, 5%, and 1% levels, respectively.

Subsequently, we proceed to ascertain the impact of digital finance on urban resilience by examining its role in facilitating industrial transformation, upgrading, as well as fostering innovation and entrepreneurship. Table 12 presents the benchmark regression results of the mediating mechanism, with industrial transformation and upgrading serving as the mechanism variable, for columns (1), (3), and (5). The findings indicate that the regression coefficient associated with digital finance exhibits a significant decrease, while the regression coefficient linked to industrial transformation and upgrading demonstrates a significant positive effect at the 1% level. This suggests that industrial transformation and upgrading serve as a mechanism through which digital finance influences urban resilience. This finding provides support for our hypothesis 2. The internal necessity to stimulate new drivers of economic development and boost economic resilience under market economic conditions arises from China's strategic reform of its economic structure, which includes guiding industry transformation and upgrading. Digital finance is a burgeoning field within financial development that is poised to play a crucial role in driving industrial transformation and upgrading. It represents a new direction and trend in the financial sector, emerging as a result of the ongoing digital technology reform and industrial digital transformation. Hence, it is imperative to proactively and steadily foster the advancement of digital finance, facilitate the profound amalgamation of digital technology and conventional financial services, accomplish the enhancement of quality and efficiency in financial services for the tangible economy, and comprehensively actualize the magnifying, duplicating, and multiplying impacts of digital technology on economic expansion.

Upon examination of columns (2), (4), and (6) in Table 12, it becomes evident that the benchmark regression outcomes exhibit a resemblance between the utilization of innovation and entrepreneurship as the mechanism variable and the utilization of industrial transformation and upgrading as the mechanism variable. The regression coefficient for digital finance exhibits a negative trend, but the regression coefficients for the core explanatory variables and the mechanism variables demonstrate statistically significant positive associations. These findings suggest that innovation and entrepreneurship play a crucial role in enhancing urban resilience. The promotion of innovation and entrepreneurship plays a crucial role in China's modernization efforts. It serves as a significant strategy to explore novel avenues and pathways for development, continuously cultivate new drivers and advantages of growth. This approach is not only pivotal in facilitating high-quality economic progress but also serves as a vital pillar in strengthening urban resilience and sustainable capacity. The findings from the regression analysis provide evidence for the presence of a mediating influence of innovation and entrepreneurship, hence supporting hypothesis 3 as posited in this scholarly work.

#### Conclusion and policy implications

This article utilizes the Beijing University Digital Finance Index data from 2011 to 2020, along with panel data of 287 cities in China at or above prefecture level. The study employs a fixed effects model and instrumental variable method to comprehensively analyze the correlation between digital finance and urban resilience. Additionally, the article empirically investigates the mechanism of innovation transformation induced by digital finance. The findings indicate that digital finance plays a crucial role in facilitating the creation of resilient cities, thereby boosting their capacity to withstand and recover from risk shocks, and ultimately fostering sustainable development. Specifically, it enhances the capacity of urban areas to adapt, adjust, and recover from periods of economic downturn and promote growth. The research of heterogeneity reveals that digital finance exhibits varying effects on urban resilience. The depth of digital finance utilization emerges as the most influential factor, followed by digitalization, and lastly, coverage. The examination of regional disparities reveals that the influence of digital finance on urban resilience exhibits notable variations across different areas. Specifically, the positive effect of digital finance on urban resilience steadily diminishes as one moves from the eastern to central to western regions. The mechanism test provides evidence that digital finance enhances the efficiency of resource allocation, mitigates limitations in financing, diminishes information asymmetry, fosters industrial transformation and advancement, and stimulates urban innovation and entrepreneurial endeavors. These outcomes collectively serve as a crucial foundation for the development of resilient cities.

The conclusions of this paper provide a research basis for understanding the development of digital inclusive finance in China and the influencing factors of urban economic resilience. At the same time, it has set a new model for the development of digital finance for cities around the world, especially for developing countries at the same stage of development to develop digital finance. First of all, all countries and regions should accelerate the construction of digital infrastructure and increase support for the development of digital finance. The prerequisite for giving full play to the advantages of digital finance such as inclusiveness, speed, and low cost, and realizing the utility value of economic growth is to establish a sound digital infrastructure. Through digital technology, a highly interconnected financial network between urban and rural areas will be built, the penetration of digital finance in various regions will be improved, the allocation of financial resources will be optimized, and the spillover effect and trickle-down effect under spatial interaction will be strengthened, so that more groups can enjoy high-quality and efficient financial services. Secondly, in countries and regions with underdeveloped formal financial systems, the development of digital finance supported by digital technology can effectively make up for the lack of financial services in the formal financial system and alleviate the prominent contradiction between supply and demand. Of course, governments should also fully recognize the differences between regional cities, introduce innovative policies to support the development of digital finance according to local conditions, and actively promote the integrated and coordinated development of digital finance and the real economy, so as to achieve sustainable urban development and enhance their own development resilience to effectively resist external shocks. Thirdly, the relevant departments should issue corresponding policy documents to create a good market environment to guide the healthy development of digital finance, and at the same time, take the initiative to strengthen financial supervision, deal with violations of laws and regulations in a timely manner, and avoid the accumulation of financial risks. Lastly, it is essential to advance education in the popularization of digital finance knowledge and augment the overall financial literacy of society. Elevating individual awareness is a pivotal prerequisite for safeguarding financial consumer rights, ensuring that citizens can partake in economic growth through accessible financial services.

## Data Availability

The data that support the findings of this study are available from the corresponding author upon reasonable request.
